# Dementia and assisted suicide and euthanasia

**DOI:** 10.1007/s00415-016-8095-2

**Published:** 2016-03-26

**Authors:** Inez D. de Beaufort, Suzanne van de Vathorst

**Affiliations:** Department of Ethics and Philosophy, Erasmus MC, PO Box 2040, 3000 CA Rotterdam, The Netherlands; Department of Ethics, AMC, Meibergdreef 9, 1105 AZ Amsterdam, The Netherlands

**Keywords:** Ethics, Dementia, End of life, Euthanasia, Assisted suicide

## Abstract

The number of dementia patients requesting euthanasia in the Netherlands has increased over the past five years. The issue is highly controversial. In this contribution we discuss some of the main arguments: the nature of suffering, the voluntariness of the request and the role of the physician. We argue that society has a duty to care for patients who suffer from dementia and to make their lives as good and comfortable as possible. We also argue that it can be morally acceptable for those who do not want to continue their life with dementia to choose to die. The choice can be based on good reasons.

## Introduction

Hugo Claus, the famous Belgian writer, chose euthanasia when he was afflicted with dementia. More famous and non-famous persons decided that it was better to end their lives than to continue to live with dementia. Dementia is a severe symptom of a number of diseases, varying from Alzheimer’s disease, Parkinson’s disease, Huntington’s disease, Lewy-body dementia, frontotemporal dementia, vascular dementia, AIDS, and OPS. Dementia is associated with problems in memory, visual-spatial orientation, language, attention, and problem solving. Dementia shortens the life expectancy, depending on the underlying cause the life expectancy after diagnosis is 3–12 years Kua et al. (14:196–201, 2014). Although some dementia patients are unaware of their own decline (anosognosia) [2], others fear the natural decline that inevitably follows the diagnosis.

Choosing death over a life with inevitable and serious decline is of all times. In the Netherlands in 2014, 81 people diagnosed with dementia opted for and were granted euthanasia, their doctors either administered them lethal drugs on their request or helped them by handing them the lethal drugs (see also Table 1).^1^

**Table 1:** Number of PAS/euthanasia for patients with dementia in the Netherlands

2014	81
2013	97
2012	42
2011	49
2010	25
2009	12

Choosing death is a morally controversial theme, helping to die even more so. And lethal help by a doctor is taboo in most countries. The Netherlands, Belgium, and Luxemburg have legislation allowing their doctors to assist at the suicide or even to administer lethal drugs on request, under specific conditions, the so-called due care criteria (see Table 2). These countries have been accused of truly sliding down the slippery slope by murdering their elderly citizens, in particular those suffering from dementia. In this contribution, we will discuss some of the central ethical arguments in the debate.

**Table 2:** The due care criteria in the Dutch Euthanasia Act

The criteria of due care of the Dutch euthanasia act require that the physician be convinced that:
There is a voluntary and well-considered request from the patient
The patient is suffering unbearably without prospect of improvement
The patient is informed about his situation and prospects
There are no reasonable alternatives to relieve suffering
An independent physician must be consulted and
Euthanasia or PAS is performed with due medical care and attention

The focus will be on the conditions for the justification of euthanasia in dementia: the issues of voluntariness (can a patient suffering from dementia make an autonomous request?), of suffering (what is the nature of suffering in dementia, can it be such that one is better off dead?), and why doctors should or should not help these patients die.

## Voluntariness

For a request for euthanasia or assisted suicide to be even considered, it is necessary that the request is made voluntary. Some will argue that no one choosing death over life can do so voluntary, because if life is so bad, there are no options left, therefore, such a choice is never made freely. We do not concur with this view on voluntariness.

There obviously is not a plethora of choices. Some frame it as a choice between life and death. Since we all die, we could also frame it as a choice of die later or die now, whereas to die later involves more suffering (Michael Stingl). To decide that this is the moment to step out, can be a voluntary choice.

However, it has been argued that those suffering from dementia suffer from a disease that itself infiltrates the very center of autonomy and voluntariness. The euthanasia cases that were notified and were judged to comply with the due care criteria in the Netherlands, therefore, in almost all cases involved persons who found themselves in the early stages of the disease. They considered themselves, and were considered by others, to be competent and to possess the capacity to decide about their death. They decided on their death at a stage of their disease when they were still able to make this autonomous choice. Having to decide, while still competent, however, may mean that people die earlier than even they might have preferred, because waiting entails the risk that they will be judged to be incompetent, and then, the opportunity will in all likelihood be gone. This is a moral problem that is not yet solved.

It is, therefore, not surprising that many people invest their hope in an advance directive. There has been a huge debate in the Netherlands on the question whether euthanasia would be justified if performed on the basis of an advanced directive, describing in detail when and under what conditions the person would want euthanasia. There has been one such a case in the Netherlands that was highly contested [3]. Though in theory, the Dutch law supports the possibility to have the request substituted by an advanced directive; in practice, this option is difficult to realize. A recently published guideline by the ministry of health illustrates this [4]. The reasons being that on the one hand, an advance directive presupposes a very careful and detailed statement on the wishes, whereas most advance directives are very general, but also that doctors find it impossible and unacceptable to perform euthanasia on a person who cannot at that moment express his or her will, nor understand what the physician is about to do, but who is present nonetheless.

Another often mentioned threat to the voluntariness of a choice for death is not related to the individual competence to decide but to the idea that there is or will be pressure from family or from society leading to feelings of a duty to die or to guilt about being alive. If the old, particularly the old suffering from diseases, are covertly or overtly (think of the Japanese minister Taro Aso [5]) given the impression that they are a burden to their families and society in general, then their requests to die will not be voluntary but more or less under pressure or even forced.

Feelings of guilt of feeling a burden toward society which the elderly (not only those with dementia) may experience depend on the respect and care society bestow on them. We hold that societies should provide good care facilities for patients with dementia. However, even in the most perfect nursing home, people will experience the end of autonomy, privacy and independence as they knew it, and have to live by other people’s schemes and rules, and in an environment with people they did not choose.

## Suffering

Why would one consider suffering from dementia so terrible that one would choose death? And is it about the suffering now or about future suffering? The argument often heard is that the suffering now is caused by the perspective of what the disease will do to one’s personality and life, and by the knowledge that it is a progressive disease the effects of which will get worse, leading to behavior changes, forgetfulness, not recognizing ones loved ones, loneliness, the feeling of being lost in one’s own life and in the mazes of one’s own mind. Many being in a state of advanced dementia are devoid of dignity. The loss of dignity, the knowledge that the lasting memory of their loved ones will be of the decomposed version of oneself, causes them to suffer now. And that is considered to be unbearable now. This is definitely the main reason for those who opt for euthanasia in the early stage of the disease.

Another part of the suffering of dementia is the pointlessness of letting nature take its course; why go on and slowly disintegrate? Why not bring a halt to the merciless process? From the moment, the diagnosis is given one can be sure that it will never get better, only worse, and that one will for certain undergo a disintegration of the self, and that will inevitably and within a definite number of years lead to death. In the course of this process, much is lost. Why would one be obliged to undergo this?

There are different arguments against these views of suffering.

## It is now or never

Considering the fear of future decline as (unbearable) suffering leads to a slippery slope. One can always imagine a future situation that is horrible, but that is not a reason to qualify the fear of, or anticipation of, the realization of such a perspective in itself as unbearable suffering. People with all kinds of diseases would then decide to choose death immediately after being diagnosed.

Against this one can argue that usually people do not want to die and, therefore, will wait and use the remaining time to live. The moment of saying ‘now it has become truly unbearable’ comes at a later stage of the disease. In the case of dementia, the nature of the decline, namely, a disintegration of one’s personality, may also be viewed as dimmer (and possibly even more frightening) than the physical decline related to other lethal diseases. And of course, the specific problem of the dementia patient is that a central characteristic of the disease is that one loses the capacity to decide about one’s fate. It is now or never. Timing is essential, and it may mean that one sacrifices some valuable time in exchange for the certainty of not having to experience further decline. Postponing is not an option as it may lead to the situation, where one cannot decide anymore and one is past postponing. Given the complications of advance directives and the understandable hesitations of doctors, deciding now implies the view that the future decline is the basis of unbearable suffering.

## You will be a different person: Alice does not live here anymore

There is an extensive philosophical debate on whether the person suffering from dementia in an advanced stage is the same person as the one at the beginning of the trajectory. If not, because there is no real continuity between the two persons as some argue, then person x at the time of diagnosis should not decide for person z later [6]. The idea is that Alice does not live in this body anymore, so we cannot allow the body of Alice—inhabited by a new Alice, or by someone completely different—to die. On the other hand, if one is convinced of the idea that there is continuity in the narrative of a person’s life and that the story is still the story of X, then this argument is not convincing. We hold that the disease through its attack on the brain turns a person not into another person but into a shadow of the previous person. What remains is not another person, but the ruins of the former person. One is not talking about ‘rebirth’ or ‘total make-over’ or ‘change’ symbolizing a newness filled with new opportunities. A dementia patient is not a new phoenix arisen from the ashes of the former person. The tragedy of the disease is its destructive nature. Many dread this disappearance of what they consider to be the essence of their individuality and personality.

## Feeding the ducks: adjust to a new kind of happiness?

However, it has been argued: if there is a chance that the later shadow seems happy and contented, enjoys life, and is taking care of, there is the long past realization of the changes in his personality and his preferences, so how can one accept choosing death before that? One might rob oneself of that contentment. We think that there is a certain danger of romanticizing life with dementia. But of course, there are people suffering from dementia who seem to be happy, or at least do not seem to suffer. The problem is that for some, an important element of the notion of suffering is precisely the idea that one might become a shadow of oneself, a person who enjoys feeding the ducks in the park, enjoys watching Teletubbies as apparently Iris Murdoch did, and sings with the nursery school nearby. The thought of becoming such a person with the loss of faculties and values and personality is precisely the nightmare. This is, however, a very personal evaluation, some do not dread such a perspective at all, but others find it horrifying and contrary to their idea of dignity. It depends on what one deems central to one’s person and to the story of one’s life, and in fact, it depends on who one is and does not want to become. Both views, albeit opposing, are personal views that deserve respect as they reflect core personal values regarding what matters.

## Life is valuable in itself

The idea of the sanctity of life, that one has no right to oppose nature and has even a duty to continue to live to the (bitter) end, or that there is value in undergoing the process of decline for yourself and/or for your significant others, is also brought forward against euthanasia or assisted suicide.

Human beings, however, continuously challenge nature and interfere with natural causes of events. Does it really imply one always has to do everything to keep people alive or stay alive? However controversial the idea of euthanasia and physician-assisted suicide may be, and many would at least agree that it is morally acceptable, even imperative, to sometimes stop, withdraw or not start a medical treatment in the best interests of the person. The term ‘natural death’ has an opaque meaning in this day and age: what is a natural death? What is so great about a natural death anyway? As for the argument that there is meaning or value in undergoing the decline, isn’t that a romanticized version of the dying process? We all need to find meaning in terrible experiences and in dying. How we do that should not be dictated by medical technology, nor by values one does not hold, nor by other people’s religious views. People differ. That may be an open door. The fact that these differences are sometimes not considered can also be (part of) the tragedy.

## Why should a doctor help?

Why should a doctor help? Doctors frequently have a role at the deathbed of their patients. It is their professional duty to make sure the dying patient is comfortable and adequately cared for. In many instances, they affect the course of events, for instance when they decide to withdraw a treatment, or to not start a treatment. A patient who dies because futile treatment was stopped or not initiated dies at that moment, because the doctor (sometimes together with the patient) made a decision that there was nothing to be gained from further treatment, even if the patient would die anyway later. The doctor is responsible, if not for bringing about the death of the patient, then for bringing death forward. In some countries, the Netherlands, for example, doctors are also permitted to help patients die on their request, under certain conditions. Whether doctors should also play a role in bringing forward the death of patients who are in principle capable of taking lethal medication themselves is contentious even in the Netherlands. Chabot argues that patients capable of this should also do it themselves, and to differentiate between this conscious act and suicide he coined the term auto-euthanasia. This can be brought about either by ingesting a lethal dose and/or combination of drugs, or by abstaining from food and drink [7]. Some patients may prefer auto-euthanasia because they do not wish to burden their physician or because they do not want euthanasia or assisted suicide. Some, however, find the prospect of having to starve yourself quite horrible, and not everybody has medication to do it oneself. There are also other arguments why people prefer their doctors to assist them in death: to make sure it all goes well (in the Netherlands, the doctor who assists in death is obliged to stay present to make sure death is calm and dignified), to make sure the right dose of the right medication is taken, and because assistance at suicide is illegal in the Netherlands if given by anyone other than a doctor, because they can die surrounded by family and friends (who would have to make sure they were absent so as not to be charged with aiding and abetting). In Oregon, doctors are not obliged to be present at the moment of ingestion, but 11 % were [8]. Depending on legal arrangements, the doctor could be present or not.

## Finally

There is no doubt to our minds that society has a duty to care for patients who suffer from dementia and to make their lives as good and comfortable as possible. There is also no doubt to our minds that it can be morally acceptable for those who do not want to continue their life with dementia to choose to die. The choice can be based on good reasons that are to a great extent very personal and intricately linked to one’s view on life and on oneself. There are different ways to bring about death. Some find themselves in a situation where they can refuse (further) treatment, others do not have that option, and for them, ‘auto-euthanasia’ or euthanasia or assisted suicide is the option they have. All options can be turned into a slippery slope scenario. We realize that for some of those who oppose euthanasia and physician-assisted suicide for patients with dementia, and the Dutch practice already is the proof of a slippery slope. Given the number of euthanasia cases compared with the number of patients suffering from dementia, we doubt it is. We all, however, have to consider the question: do people who do not want to experience the further decline have to hope for a life threatening infection or will other options be made available, and how can we then best protect people in such a way that the choice to die is theirs and theirs only?

## Footnotes

1 Assisted suicide or euthanasia is also possible in Belgium, but Belgian statistics specific for dementia are not available.
